# Vegetation and carbon sink response to water level changes in a seasonal lake wetland

**DOI:** 10.3389/fpls.2024.1445906

**Published:** 2024-11-05

**Authors:** Weiyu Huang, Xin Liu, Lin Tian, Geng Cui, Yan Liu

**Affiliations:** ^1^ College of Geographic Sciences, Changchun Normal University, Changchun, China; ^2^ Institute of Water Resources Engineering, Changchun Institute of Technology, Changchun, China; ^3^ State Key Laboratory of Black Soils Conservation and Utilization, Northeast Institute of Geography and Agroecology, Chinese Academy of Sciences, Changchun, China

**Keywords:** water level, Hongze lake, gross primary productivity, net plant productivity, fractional vegetation cover, google earth engine

## Abstract

Water level fluctuations are among the main factors affecting the development of wetland vegetation communities, carbon sinks, and ecological processes. Hongze Lake is a typical seasonal lake wetland in the Huaihe River Basin. Its water levels have experienced substantial fluctuations because of climate change, as well as gate and dam regulations. In this study, long-term cloud-free remote sensing images of water body area, net plant productivity (NPP), gross primary productivity (GPP), and Fractional vegetation cover (FVC) of the wetlands of Hongze Lake were obtained from multiple satellites by Google Earth Engine (GEE) from 2006 to 2023. The trends in FVC were analyzed using a combined Theil-Sen estimator and Mann-Kendall (MK) test. Linear regression was employed to analyze the correlation between the area of water bodies and that of different degrees of FVC. Additionally, annual frequencies of various water levels were constructed to explore their association with GPP, NPP, and FVC.The results showed that water level fluctuations significantly influence the spatial and temporal patterns of wetland vegetation cover and carbon sinks, with a significant correlation (P<0.05) between water levels and vegetation distribution. Following extensive restoration efforts, the carbon sink capacity of the Hongze Lake wetland has increased. However, it is essential to consider the carbon sink capacity in areas with low vegetation cover, for the lakeshore zone with a higher inundation frequency and low vegetation cover had a lower carbon sink capacity. These findings provide a scientific basis for the establishment of carbon sink enhancement initiatives, restoration programs, and policies to improve the ecological value of wetland ecosystem conservation areas.

## 1 Introduction

Wetlands account for 2%–6% of the world’s total land area and comprise one-third of the soil organic carbon (SOC) resources. They play an irreplaceable role in regulating water sources, controlling runoff, purifying water, sequestering carbon, releasing oxygen, improving the climate, recycling nutrients, maintaining the ecological balance of the region, and providing biological habitats (Kayranli et al., 2010; Ren et al., 2022). Carbon emissions have emerged as a growing concern due to global warming. Wetlands play an important role in the global carbon stock, serving as crucial carbon sinks owing to their low organic matter decomposition rates and high productivity (Gorham, 1991). Wetland vegetation plays an important role in carbon sequestration (Yisen et al., 2023). A decrease in vegetation can lead to an increase in greenhouse gas emissions. When soils are exposed to sunlight, the surface temperature increases, oxidation is enhanced, decomposition of organic matter intensifies, and eutrophication accelerates. This results in an imbalance in biomass and plant growth; both in the leaves and roots, which alters the accumulation of organic matter and carbon. Therefore, the carbon sink function of wetlands can be directly or indirectly affected by plant productivity (Ballut-Dajud et al., 2022).

Although the mean water level of wetlands has been significantly and positively correlated with the waterbody area ratio (Yang et al., 2023; Wang et al., 2024, 2023), wetland hydrology remains poorly understood, partially because relatively few tools are available for monitoring and assessing them at the landscape scale (Halabisky et al., 2016). In recent years, researchers have proposed various indices, such as the modified normalized difference water index (MNDWI) and normalized difference water index (NDWI), and techniques, such as the Dynamic Surface Water Extent (DSWE) angular observation of SAR, to understand changes in wetland waters. This indicates that data combining optical and SAR multisensory approaches to measure the extent of inundation are often more reliable (Acharya et al., 2016; Ahmad et al., 2019; Brakenridge and Anderson, 2006; Chen M., et al., 2023; Jones, 2019; McFeeters, 1996; Xu, 2006). Wetland hydrological processes drive the evolution of wetland vegetation cover and plant community composition. This has resulted in changes in wetland carbon sink patterns. To address the impacts of different hydrological elements on wetland vegetation, researchers have studied the fractional vegetation cover (FVC) of wetlands at different water levels. They found that the increase and decrease in water level had significant effects on the FVC and evapotranspiration (Chen W., et al., 2023; Liu et al., 2021; van der Valk et al., 1994; Zheng et al., 2021). In studies on the distribution patterns of wetland vegetation, researchers have investigated the spatiotemporal dynamics of vegetation in wetlands, such as estuarine wetlands, seasonal lakes, and diffuse lakes. The spatial heterogeneity of vegetation extent and its response to water level fluctuations and microtopography has also been examined to assess the effects of different water level conditions and topographic elements on wetland vegetation distribution (Peng et al., 2022; Huang et al., 2023; Zhang C., et al., 2022). The spatial and temporal patterns of inundation of lakes and floodplain wetlands have also been investigated. Floodplain wetland heterogeneity and its effects on wetland vegetation have been studied (Huang et al., 2023). This suggests that changes in inundation patterns due to lake wetlands may inhibit downward expansion of vegetation and promote vegetation expansion to higher elevations (Huang et al., 2023). The effects of seasonal water level fluctuations on wetland vegetation have also been examined, and it has been found that water level fluctuation (WLF) is a vital natural process for the sustainability of different plants and it plays a crucial role in maintaining productivity and wetland biodiversity (Dai et al., 2019). However, to date, relatively few studies have explored the response of carbon sequestration elements in seasonal lake wetland vegetation to hydrological conditions.

Gross primary productivity (GPP) is the total amount of carbon fixed by terrestrial ecosystems via vegetative photosynthesis. The normalized difference vegetation index (NDVI) can be used to assess the chlorophyll content effectively. It is an accurate representation of vegetation density parameters, such as FVC, and the fraction of absorbed photosynthetically active radiation. Therefore, the NDVI is strongly correlated with FVC and GPP (Camps-Valls et al., 2021; Westergaard-Nielsen et al., 2013). Monitoring changes due to natural and anthropogenic impacts is important for assessing the carbon cycle (Fang et al., 2018). Net plant productivity (NPP) is the reference for determining carbon sources and sinks. Therefore, it is a core component of the wetland ecosystem carbon budget (Zhang M., et al., 2022; Cloern et al., 2021). Currently, there are various methods to study GPP, NPP, and FVC. This includes combining remote sensing technology, ecological modeling, and field observations. Some researchers have used remote sensing technology to determine the influence of spatial and temporal variations on NPP, GPP, and vegetation cover in wetlands (Yang et al., 2023; Zhang M., et al., 2022; Kang et al., 2023). In addition, some researchers have applied the Carnegie–Ames–Stanford Approach (CASA) model and the Boreal Ecosystem Productivity Simulator (BEPS) model to evaluate and validate the wetland NPP (He et al., 2021). However, the simulation results for certain ecological processes may also be subject to a degree of uncertainty. Real vegetation growth data can be directly obtained through field observations and combined with other data to verify and calibrate monitoring results (Gayet et al., 2022; Alam et al., 2024). However, this requires considerable labor, materials, and time. For the estimation of GPP and NPP and the analysis of spatial and temporal patterns, scholars have processed and analyzed the coastal wetlands in the floodplain of Dongting Lake and the mouth of the Yangtze River in China using the eddy covariance technique, and Landsat and MODIS satellite data, and found that vegetation and water level were the dominant factors affecting GPP (Yang et al., 2023; Xu et al., 2018a). The spatial and temporal patterns of NPP have been analyzed using long-term satellite data for wetlands in China, such as swamp wetlands in the Yellow River source area, and wetlands in Inner Mongolia. These large-scale regional analyses indicate that natural drivers, such as climate conditions, vegetation type, land cover type, precipitation and temperature, are positively or negatively correlated with the NPP of wetland vegetation (Ren et al., 2021; Tian et al., 2023; Zhang et al., 2023; Liu et al., 2022). Similarly, spatial and temporal analyses of NPP and its relationship with climatic factors in Asian regions, such as Iran and the west-central Korean Peninsula, have been conducted, and precipitation frequency has been found to be the dominant climatic factor influencing the annual variation in NPP (Kamali et al., 2020; Kang et al., 2005). In addition, some scholars from European countries, such as Spain and Germany, analyzed the relationship between NPP and environmental factors in time and space, and found a strong association between the total annual NPP and the distribution of land cover types (Gingrich et al., 2015; Ma, 2020; Vernet et al., 2021; Martínez et al., 2022).

The Hongze Lake wetland is a typical lake wetland in the Jianghuai region, located in the middle and lower reaches of the Yangtze River. Fluctuations in the wetland water levels before the 1950s were mainly driven by nature. In the last 70 years, anthropogenic activities, especially hydraulic engineering, have had a considerable impact on the hydrological situation of the lake, particularly lock-and-dam operations and agricultural irrigation. This led to a substantial reduction in the environmental flow component (EFC). Manipulation of locks and dams during the dry season has a substantial impact on the hydrology of the lake, with changes of 37% and 41% in water levels, respectively (Wei et al., 2024; Yin et al., 2013). The water level of Hongze Lake is mainly influenced by irrigation and water conservation projects. The area is dominated by lakeshore reclamation and aquaculture activities (Cai et al., 2020). Since the establishment of the Hongze Lake National Nature Reserve (HLNNR) in 1999, restoration construction projects have been implemented. After the implementation of buffer zone resettlement and seine removal projects, a majority of the HLNNR area was restored to its early ecosystem status. Prior research has been conducted on the restoration of HLNNR ecosystems from the perspectives of meteorological factors, soil moisture, community composition, vegetation types, and vegetation planting programs, using remote sensing and location monitoring data (Ji et al., 2021; JIANG et al., 2023; Qiu et al., 2020; Wang et al., 2022; Wu et al., 2018; Xu et al., 2018b). Measurements of carbon fluxes, soil alkaline phosphatase kinetic characteristics, soil microbial characteristics, enzyme activities, and other related indicators have been used to determine soil organic carbon quality and carbon sink functions in HLNNR (Xu et al., 2018a; Xu et al., 2018b; Xu et al., 2020; Cui et al., 2022; Zhao and Cao, 2018).

However, research on the carbon sequestration functions of HLNNR remains limited. The main objectives of this study were to: (1) calculate the spatial and temporal characteristics of hydrological changes, vegetation distribution, and carbon sequestration indicators of the HLNNR from 2006 to 2023; (2) examine the intrinsic connection between the hydrological conditions and vegetation distribution patterns of the Hongze Lake wetland; and (3) determine the main hydrological elements that affect the carbon sink capacity of wetland vegetation.

## 2 Materials and methods

### 2.1 Study area

**Figure 1:**
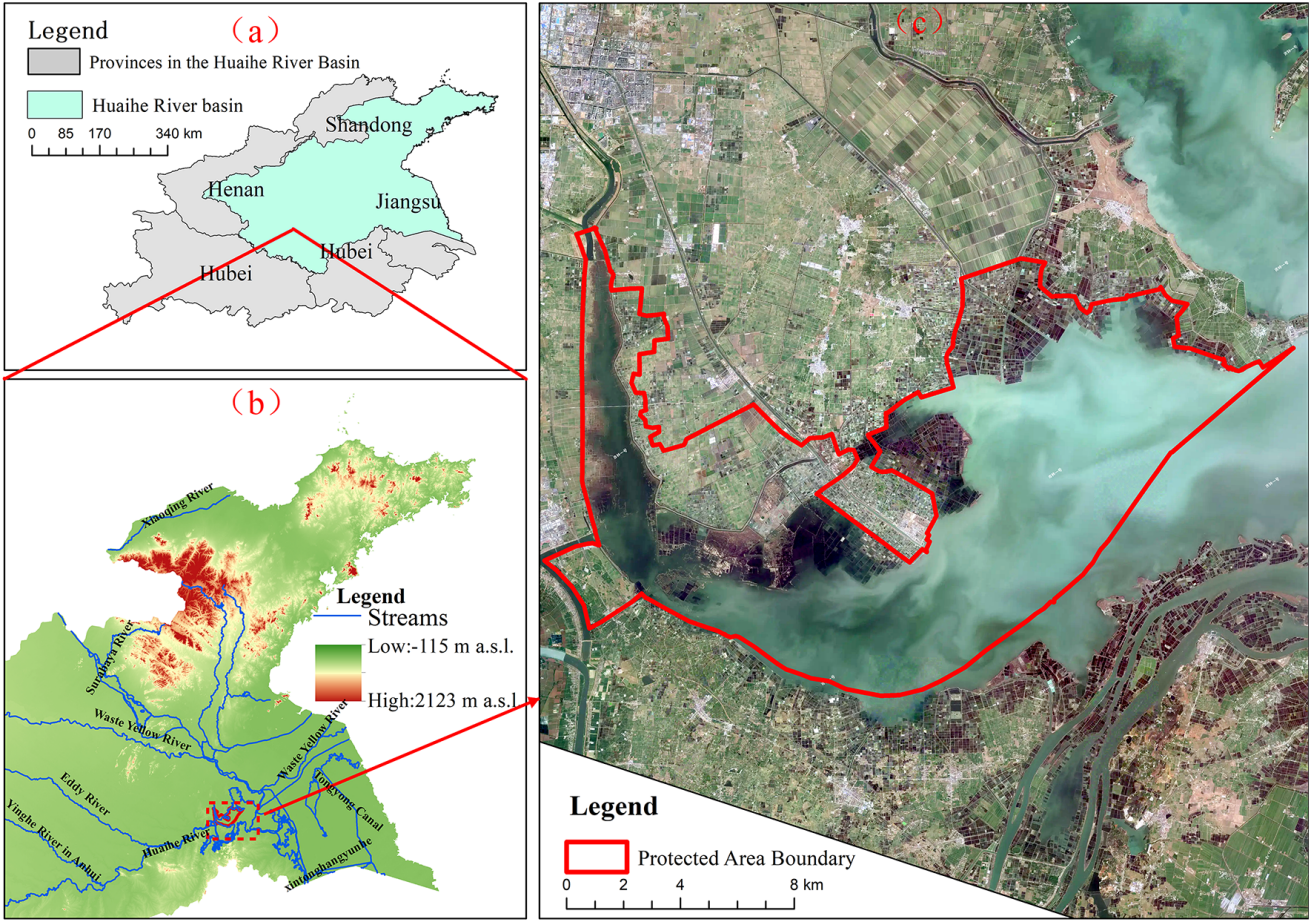
Location of the study area. **(A)** Location of Huaihe River Basin, China; **(B)** location of HLNNR in Hongze Lake; **(C)** boundary of HLNNR. Reprinted with permission from Jilin-1 Satellites by Chang Guang Satellite Technology Co., LTD., https://www.jl1mall.com.

HLNNR is located in the northwestern part of Hongze Lake in Jiangsu Province, and is also the estuary area where the HuaiheRiver enters Hongze Lake, which is classified as a mudflat water areaunder the inistrative jurisdiction of Sihong County (**Figure 1**). The study area ranges from 33°09′13.475′′N to 33°23′06.796′′N and 118°11′23.496′′E to 118°38′13.891′′E. The wetland is located between the northern subtropical Jiangbei District and the southern temperate Luhuai District, which has monsoon climate characteristics and is subject to the regulating influence of the surface of Hongze Lake. The climate of the lake is governed by the monsoons and the area has four distinct seasons. The wetland drains 158,100 km^2^ of water from the upper and middle reaches of the Huaihe River. The rivers entering the lake mainly include the main channel of the Huaihe River, the Huaihong New River, the Xinbian River, the Xuhong River, and 17 other rivers, among which the main channel of the Huaihe River accounts for more than 70%. Rivers outside the lake include the waterways into the river, the general irrigation canal of North Jiangsu Province, and the waterways into the sea. These are mainly distributed in the eastern part of the lake. Since the 1980s, there has been remarkable growth and development in circle dike construction, purse seine aquaculture, and fishing industries around the Hongze Lake wetland. Habitat fragmentation and species homogenization in the Hongze Lake wetland are becoming increasingly severe issues. Since being designated as a national nature reserve in 2006, the management of the HLNNR area has prioritized addressing issues such as the shrinking of wetland areas, fragmentation of habitats, deterioration of the aquatic environment, and reduction of species richness caused by large-scale purse seine (dike) farming, lake cultivation and fishing activities. The HLNNR focuses on restoring ecosystems, including wetlands, and transferring production and employment. Substantial financial and human resources have been allocated towards effective management, leading to substantial reduction in fragmentation in the HLNNR area.

### 2.2 Data sources and processing

#### 2.2.1 Water level data

WLF and alternating changes in inundation time are key hydrological processes in overwater lakes. In this study, data such as daily water levels at hydrological stations from 2006 to 2023 were obtained from the Water Information Network of Jiangsu Water Resources Department, Jiangsu, China, and the National Earth System Science Data Center (https://www.geodata.cn).

#### 2.2.2 Remote sensing data for water bodies

Acquiring a water body for remote sensing involves constructing an annual median image of the water body by using the GEE platform. The process is as follows. (1) Choice of data source: The water body data source is the JRC Monthly Water History v1.4(Source: EC JRC/Google), a database that has global water body distribution maps from 1987 to 2021 extracted from Landsat data. (2) The timeframe of the data was 2006–2023 and the location screen was the HLNNR. (3) Criterion for a body of water (a pixel is a body of water if 7 months of the pixel is water). (4) Image data export: The resolution was set to 30 m × 30 m. (5) The areas of the water bodies in the HLNNR were counted. (6) The time-iteration function was then run. 7) The complete study time interval was supplemented with water body data for 2022 and 2023, using the sentinel-2 dataset. The JRC Global Surface Water Mapping Layers v1.4(Source: EC JRC/Google), a complete dataset (Ren et al., 2021), was used to analyze the changes in water resource distribution. A map of the location and temporal distribution of surface water bodies in the study area was constructed. (8) Finally, the raster layout was processed using ArcGIS10.8 software.

#### 2.2.3 Vegetation remote sensing data

Vegetation data were obtained from Landsat 5 SR Tier 1 (2006–2011), Landsat 7 SR Tier 1 (2012), Landsat 8 SR Tier 1 (2013–2018), and Sentinel 2 (2019–2023). Four datasets from the GEE dataset were provided by the US Geological Survey (USGS). Landsat 5, 7, and 8 were geometrically refined, radiometrically calibrated, and atmospherically corrected using the LaSRC (Huang et al., 2021). The spatial resolution of the land and sentinel remote sensing images selected for this study was 30 m × 30 m.

#### 2.2.4 NPP and GPP remote sensing data

The estimation of vegetation carbon stocks and sinks in the HLNNR was based mainly on the MODIS/061/MOD17A3HGF dataset (ASTER GDEM is a product of Japan’s Ministry of Economy, Trade, and Industry (METI) and NASA. Projects that are continuing to use ASTER GDEM Version 2 data are required to continue following these redistribution and citation requirements). This provides information on the annual GPP and NPP at a resolution of 500 m pixels. The GEE platform was used to define the data area, export the NPP and GPP data year-by-year from 2006 to 2023, and convert the data units to kg/m^2^ to export the image dataset to Google Drive. The average NPP and GPP values per square in the study area were statistically analyzed using simple functions.

#### 2.2.5 Construction of different water level parameters

Annual daily water level observations at the Jiangba Hydrological Station were used to analyze the characteristics of the water level changes. The annual maximum water level (max), annual mean water level (mean), annual minimum water level (min), and the overall variance parameter of the effective water level during the year were extracted to determine the strength of the WLF rate. When the water level of Hongze Lake reached 11.9 m (m above sea level), the aquatic vegetation gradually became inundated and was completely inundated during the flood season from April to October. According to the ecological and economic analysis and social risk assessment, the water level above 13.6 m at Jiangba Hydrological Station was identified as the “warning level” by the Hydrological Bureau of Jiangsu Province. According to the warning and vegetation inundation levels, different water level gradients (L11.95, L12.05, L12.15, L12.25, L12.35, L12.45, and L12.55) were set to extract the percentage of days in a year that exceeded these levels. The WLF parameter sets are listed in **Table 1**.

**Table 1:** Parameter abbreviations and their descriptions.

Parameters	Descriptive
max	Maximum daily effective water level throughout the year
mean	Average of daily effective water levels throughout the year
min	Minimum daily effective water level throughout the year
WLF	The total variance of the effective water level for each day of the year, characterizing the annual WLF rate
L11.95	Percentage of days with effective water level data exceeding 11.95 m for the year
L12.05	Percentage of days with effective water level data exceeding 12.05 m for the year
L12.15	Percentage of days with effective water level data exceeding 12.15 m for the year
L12.25	Percentage of days with effective water level data exceeding 12.25 m for the year
L12.35	Percentage of days with effective water level data exceeding 12.35 m for the year
L12.45	Percentage of days with effective water level data exceeding 12.45 m for the year
L12.55	Percentage of days with effective water level data exceeding 12.55 m for the year

#### 2.2.6 Fractional vegetation cover calculation

To reflect the growth status of wetland vegetation more effectively, we used the GEE platform to construct a median annual image collection of FVCs, calculate the regional average data, and classify the FVC. The calculation process is as follows: (1) To construct a long-term dataset, we first input the three-dataset series of remote sensing images into the GEE code editor and then performed preprocessing, such as temporal filtering, cloud totaling, and deletion. (2) The images in the dataset of Step 1 were grouped annually, the median value of each pixel of the images in the group was calculated, and an annual median image dataset was generated. (3) The NDVI was calculated using the normalized difference function of the dataset in Step 2 to generate a median annual NDVI image set. (4) The mask function was used to remove water bodies to avoid the impact of excessive water body area on vegetation cover calculation, specifically masking image data values less than zero to generate NDVI images. 5) The FVC was calculated using the pixel binary modeling approach by selecting the cumulative frequency of the image set obtained in Step 4. Here, 5% and 95% of the NDVI values were used as NDVIsoil and NDVIveg, respectively, to generate an annual median FVC image set. (6) Using the study area vector data and dataset from Step 5, the average FVC value of the study area was calculated by calling the simplified area function. The area of each FVC classification feature class was calculated as shown in **Table 2**. (7) The image dataset was exported from Step 5 to Google Drive with a resolution of 30 m × 30 m. (8) FVC classification maps were generated based on **Table 3**, and the FVC was reclassified into five classes using ArcGIS.

**Table 2:** Classification and characterization of vegetation cover.

FVC categorization feature level	FV (%)	Classification features
Low FVC	0–20	The surface is largely devoid of vegetation, bare soil, bare rock, and water
Lower FVC	20–40	Sparse vegetation
Medium FVC	40–60	Medium vegetation
Higher FVC	60–80	Dense vegetation
High FVC	80–100	Denser vegetation

**Table 3:** sen + MK test trend categories.

Slope	Z	Trend type	Trend features
*Slope*>0	2.58<Z	4	Dramatically increased
	1.96<Z ≤ 2.58	3	Significant increased
	1.65<Z ≤ 1.96	2	Slightly significantly increased
	Z ≤ 1.65	1	No significant increased
*Slope* = 0	Z	0	Stable
*Slope*<0	Z ≤ 1.65	−1	No significantly reduced
	1.65<Z ≤ 1.96	−2	Slightly significantly reduced
	1.96<Z ≤ 2.58	−3	Significantly reduced
	2.58<Z	−4	Dramatically reduced

#### 2.2.7 Vegetation cover trend analysis

A combination of the Theil–Sen median trend analysis (Equation 1) and Mann–Kendall (MK) test (Equation 2) was used to determine trends in vegetation for the long time-series data. The advantages of this method include not requiring data to adhere to a specific distribution, demonstrating strong resistance to data errors, and being supported by a solid statistical theoretical basis for testing the level of significance. This enhances the scientific validity and credibility of the findings (Ahmed, 2014).


(1) $$\begin{array}{c} Slope\;=\;Median\;\left(\frac{FVC_{j}-FVC_{i}}{j-i}\right),2006\leq i<j\leq 2019 \end{array}$$

where FVCj and FVCi are the data values at times j and i (j > i), respectively.

Median () represents the median value, slope > 0 indicates that the FVC is in a growing trend, and slope < 0 indicates that the FVC is degrading.

The MK test is as follows:


(2) $$\begin{array}{c} \;S=\sum_{i=1}^{n-1}\sum_{j=i+1}^{n}sgn\left(\;FVC_{j}-FVC_{i}\right),2006\leq i<j\leq 2023 \end{array}$$

where n is the number of observations, and FVC_j_ and FVC_i_ are the numbers of observations at time j and time i, respectively.

Using sgn (FVCj-FVCi) as a sign function, according to the magnitude of the FVCj-FVCi value, 1, 0, and −1 are defined as increased, unchanged, and decreased, respectively (Equation 3):


(3) $$\begin{array}{c} sgn\;\left(FVC_{j}-FVC_{i}\right)=\{\begin{array}{c} 1,\;FVC_{j}-FVC_{i}>0\\ 0,\;FVC_{j}-FVC_{i}=0\\ -1,\;FVC_{j}-FVC_{i}<0 \end{array} \end{array}$$

In this study, because the sample size was >10, the variance of S was calculated as follows (Equation 4):


(4) $$\begin{array}{c} Z=\{\begin{array}{c} \frac{S-1}{\sqrt{Var\left(S\right)}},S>0\\ 0\;,S=0\\ \frac{S+1}{\sqrt{Var\left(S\right)}},S<0 \end{array} \end{array}$$

The statistical formula for the standardized z-test data for a normal standardized distribution (Equation 5).


(5) $$\begin{array}{c} \text{Var}\left(S\right)=\frac{n\left(n-1\right)\left(2n+5\right)}{18} \end{array}$$

These steps were performed in MATLAB (**Table 3**) to complete the trend significance of the assessment, the trend analysis of vegetation cover, and significance test results for reclassification. This was conducted using ArcGIS 10.8 software to overlay data, and to identify trends of change and assess the significance of the coupling results.

## 3 Results

### 3.1 Hydrological pattern changes

#### 3.1.1 Water level changes

**Figure 2:**
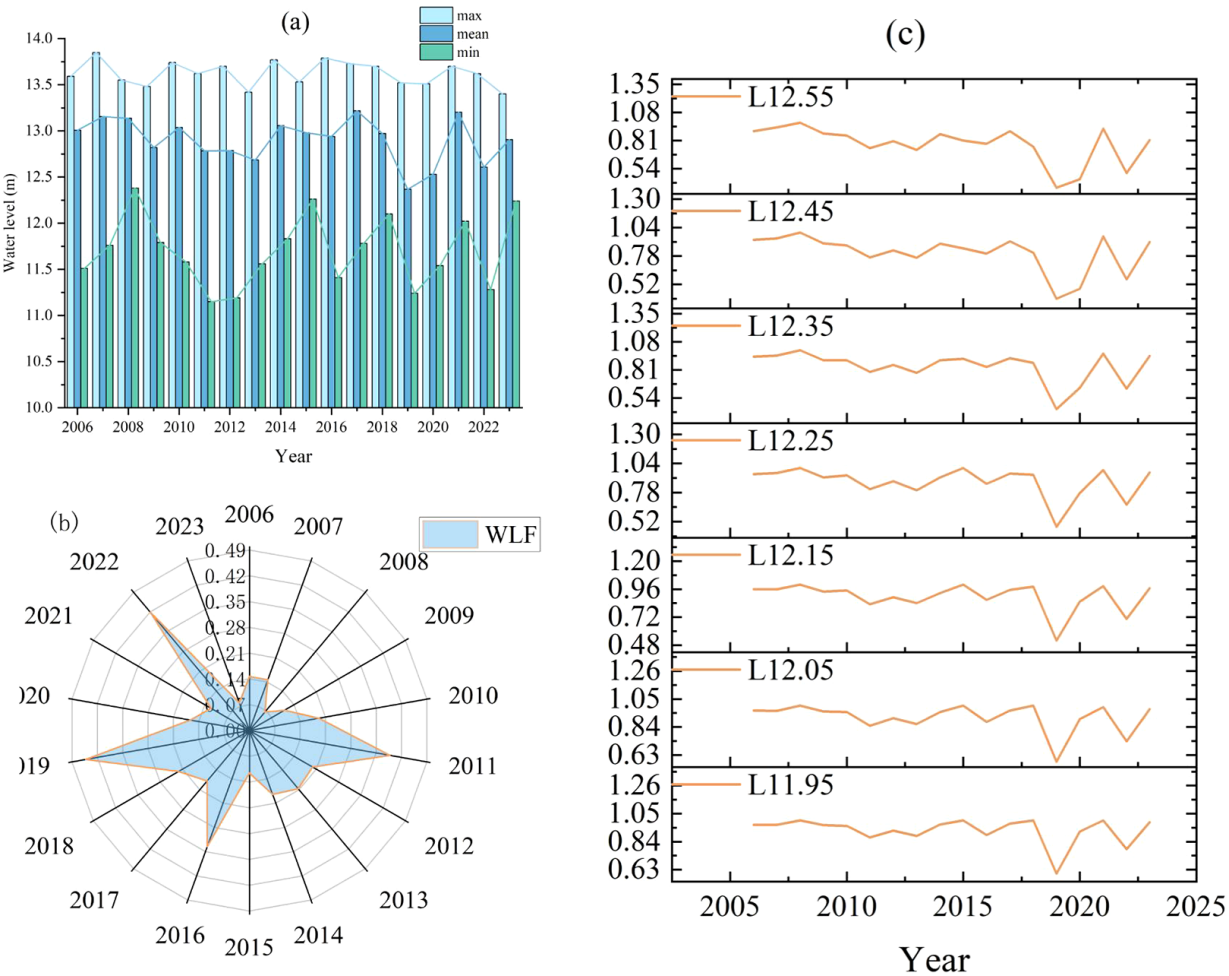
Fluctuation of water level parameters. **(A)** max, mean, and min; **(B)** WLF; **(C)** L11.95, L12.05, L12.15, L12.25, L12.35, L12.45, and L12.55.

As changes in water levels have different effects on aquatic vegetation, we used the nine parameters shown in **Figure 2** to describe the changes in water levels from 2006 to 2023. The trends of max, mean, and min were the same, with multi-year averages of 13.62, 12.90, and 11.70 m, respectively (**Figure 2A**). Among them, the maximum value for max was 13.85 m in 2007, which exceeded the warning level of 13.6 m at Jiangba Hydrological Station. Flood events occurred more frequently in all years, except for 2006, 2008, 2009, 2013, 2015, 2019, 2020, and 2023, when flooding events exceeded the warning level. In 2006, 2010–2013, 2016, 2019, 2020, and 2022, there were min values below 11.6 m. This low-water event in Hongze Lake can lead to an ecological water deficit for aquatic plants. During the study period, the maximum WLF value in 2019 was 0.45. The minimum WLF value in 2008 was 0.065, with a mean value of WLF was 0.27, and a range of variability of 0.385. The WLF has a large amplitude without a significant oscillatory cycle owing to the influence of meteorological conditions and hydrological processes, such as annual precipitation, annual evapotranspiration, and incoming and outgoing conditions (**Figure 2B**). The trend for L11.95–L12.55 was the same. There was a significant decrease in the number of days when the water level exceeded L11.95–L12.55 during the years 2019 and 2022. This indicates that the water level was generally lower during these two years (**Figure 2C**). Overall, there were substantial and complex wetland water level changes.

#### 3.1.2 Changes in water body distribution

**Figure 3:**
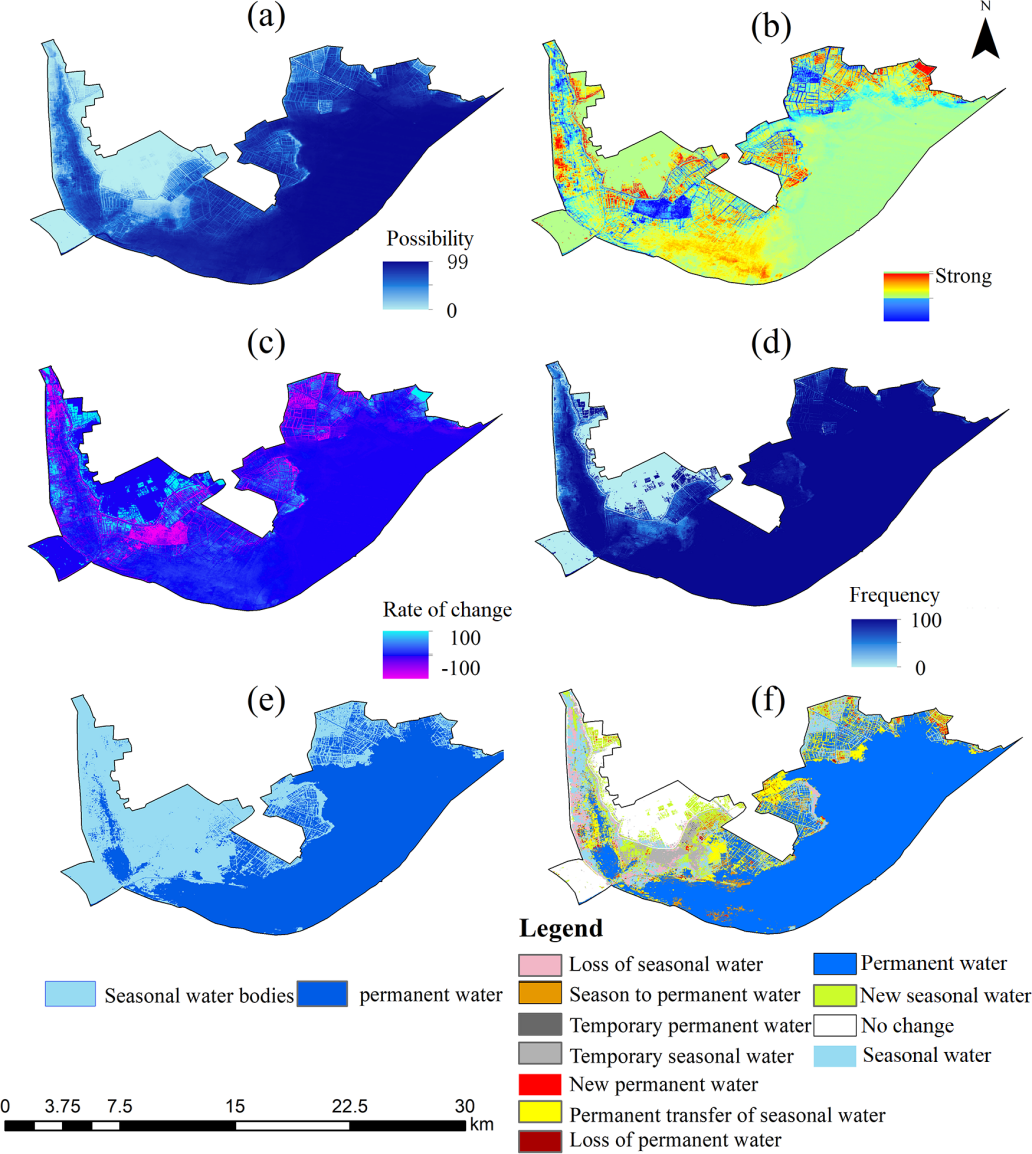
Changes in surface water bodies. **(A)** Distribution of water bodies; **(B)** Surface water intensity; **(C)** Rate of water body change; **(D)** Return frequency; **(E)** Seasonal situation; **(F)** Seasonal variation.

**Figure 4:**
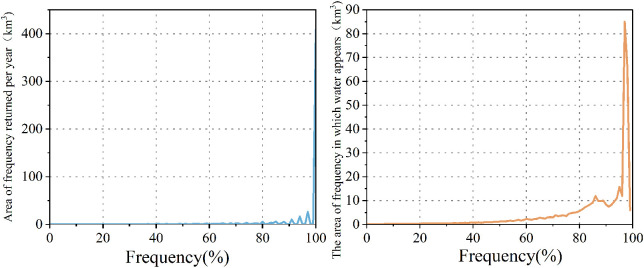
Frequency of occurrence of water bodies and frequency of recurrence of water bodies in the study area from 1984 to 2021.

**Figure 5:**
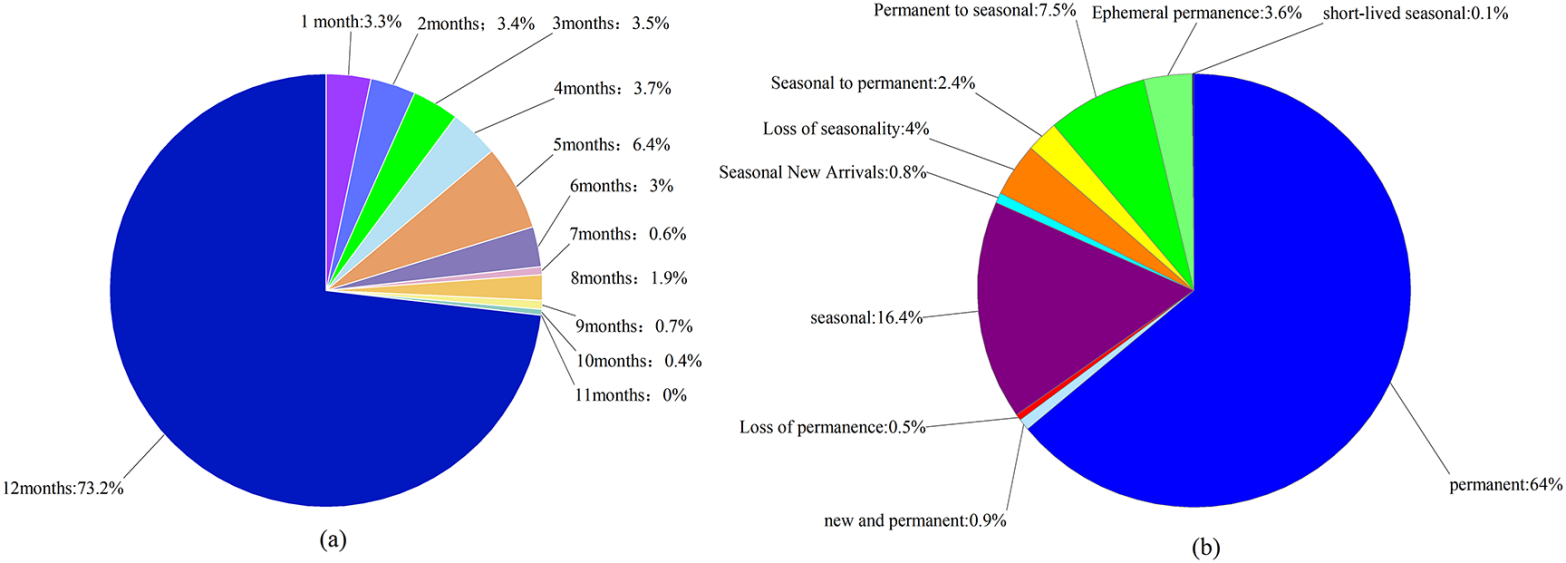
HLNNR Changes in waterbody transitions **(A)** and months of waterbody presence **(B)** from 1984 to 2021.

The results of the GEE interpretation show the changes in the distribution of water bodies in the HLNNR from 1984 to 2021. The frequency of water presence results showed that water was consistently present in the southeastern portion of the wetland, throughout the study period. This was in contrast to the northern portion of the area, where the probability of water presence was not as high (**Figure 3A**). **Figure 3B** shows the absolute change in the water bodies over the study period, that is, an increase in water in positive areas and a decrease in water in negative areas. This indicates a marked increase or decrease in water levels, except in the watershed southeast of the wetland and a small portion near the north. Similarly, substantial changes in the presence of surface water, except for the waters southeast of the HLNNR and a small portion near the north, showed a lack of normalized change in the overall water bodies (**Figure 3C**). **Figures 3D**, **4** illustrate the annual frequency of water returns in the HLNNR. The highest percentage of water return originates from the estuary and flows to the central and eastern regions, suggesting that these regions experienced consistent water recovery over time. In contrast, there was no notable consistency in the frequency of water return annually, except for the estuary and the central and western regions. **Figure 3E** shows the number of months in which water bodies were present in the HLNNR. A period of 12 months was considered permanent water, and less than 12 months was considered seasonal water. The data shown in **Figures 3E**, **5A** indicate that the presence of December water bodies is common in the study area, with an area percentage of 73.18%. This can be attributed to most of the waterbodies in the southeastern portion of the wetland being permanent. The consistently increasing water body area from January to May can be attributed to the higher precipitation in the HLNNR region during January–May.

The HLNNR’s classification of water resources changed markedly from 1984 to 2021 (**Figures 3F**, **5B**). Of these, 64% were identified as having a permanent water cover. Of this permanent water, 0.9% was newly classified as permanent but was reduced by another 0.5%. In addition, 16.4% of the HLNNR was detected as a seasonal water resource. There was a 4% loss of seasonal water resources, and 0.8% was added as seasonal water resources. There was a 2.4% increase in the conversion of seasonal water resources to permanent water resources in the HLNNR. However, 7.5% of the permanent water in the study area was converted into seasonal water during this period. **Figures 3F**, **5B** shows that 3.6% and 0.1% of the water resources in the HLNNR were categorized as transient permanent and transient seasonal, respectively. Overall, there were substantial changes in water resources in the HLNNR.

**Figure 6:**
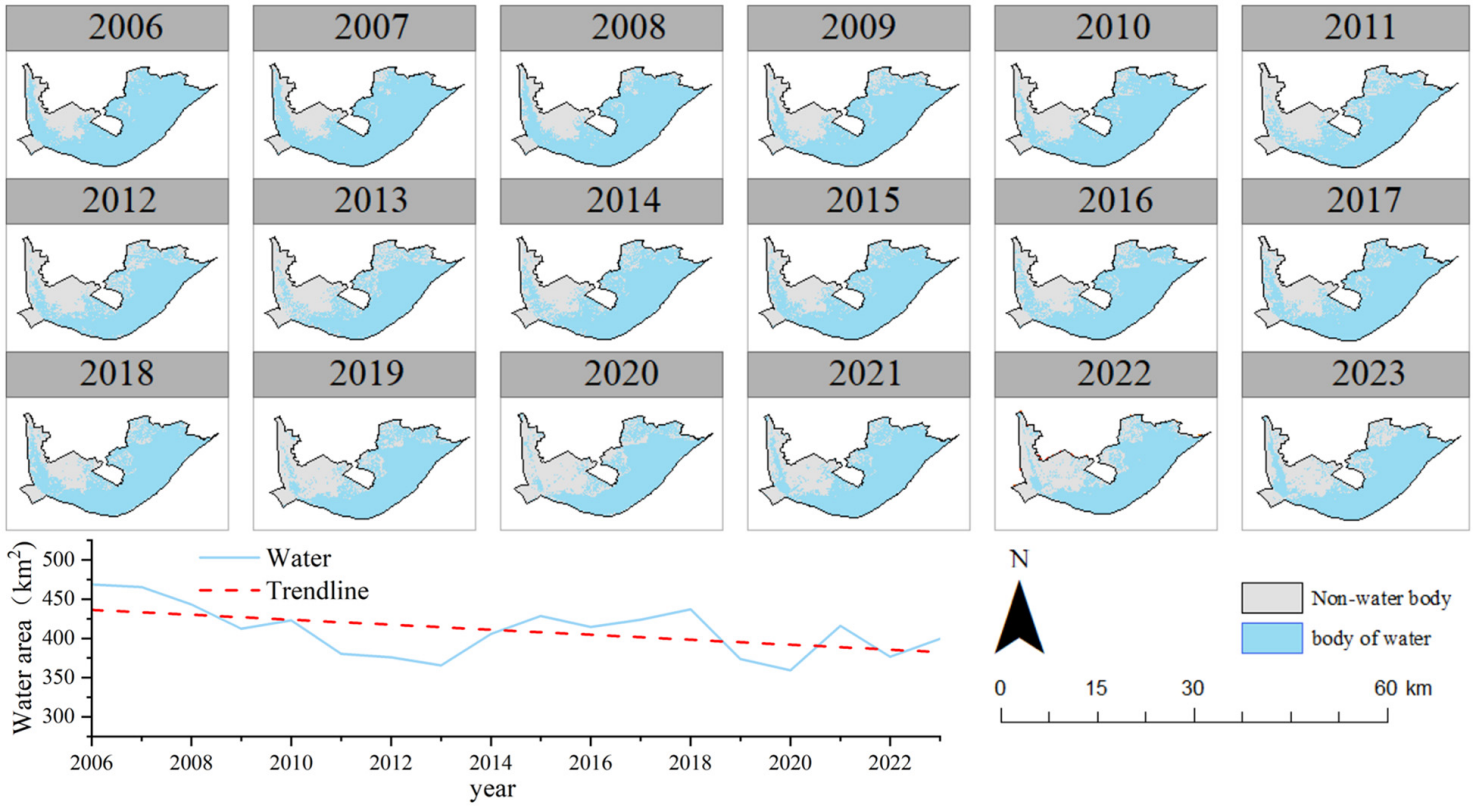
Spatial pattern and inter-annual variability of water body areas.

The area distribution of the HLNNR water bodies during the study period is shown in **Figure 6**. This shows an overall decreasing trend in HLNNR water bodies. The overall fluctuation range was 359.43 km–468.70 km^2^, with an average water body area of 409.43 km^2^ and a variance of 0.002. It reached its highest value in 2006, accounting for 76.63% of the total area, and its lowest in 2013, accounting for 60.51% of the total area. The fluctuation in the average water body area value from 2006 to 2023 was relatively small. The maximum increase or decrease did not exceed 13.8% of the average value of the water body area.

### 3.2 Spatial and temporal changes in vegetation distribution

#### 3.2.1 Time-series changes in vegetation cover

**Figure 7:**
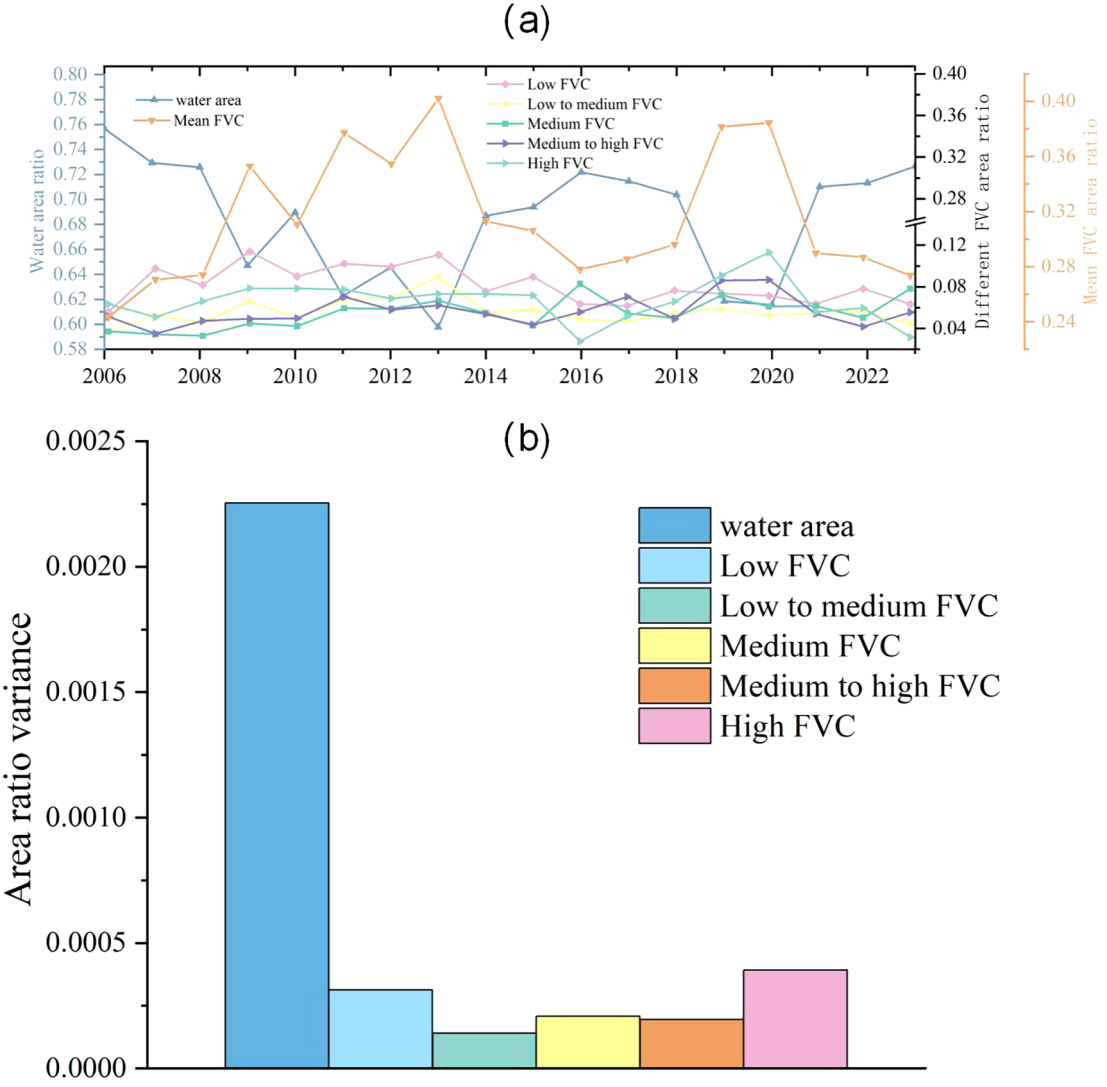
Time series changes of water body surface and FVC area **(A)** and variance of different levels of FVC **(B)**.

During the study, the average vegetation cover area ratio of the HLNNR peaked at 39.5% in 2013, with a minimum value of 23.4% in 2023 and a mean value of 31.6%. (**Figure 7A**). The number of years with a high area ratio of high FVC and a high area ratio of medium FVC was 11.1% for the entire study period. The number of years with a high percentage of low FVC was 66.6% during the entire study period. The lowest percentage was 3.47% of the total area in 2006, which was more stable than that in the other areas. The mean vegetation cover area ratio and water body area ratio showed opposite fluctuating trends, indicating that the reduction in water body area promoted vegetation growth. Meanwhile, the increase in water body area compressed the space for aquatic vegetation growth, resulting in a reduction in vegetation cover area.

**Figure 7B** shows the variance of the HLNNR multi-year watershed area ratio and vegetation cover area ratio. The higher vegetation cover ratio had a variance of 0.00039, which was significantly higher than the area ratios of the other levels of cover (p<0.05). This indicates that areas with higher vegetation cover are more sensitive to changes in the water bodies. This is due to their presence in areas with greater water depths, and even a slight increase in the water level can exceed the ecological threshold for vegetation.

#### 3.2.2 Spatial pattern of vegetation cover

**Figure 8:**
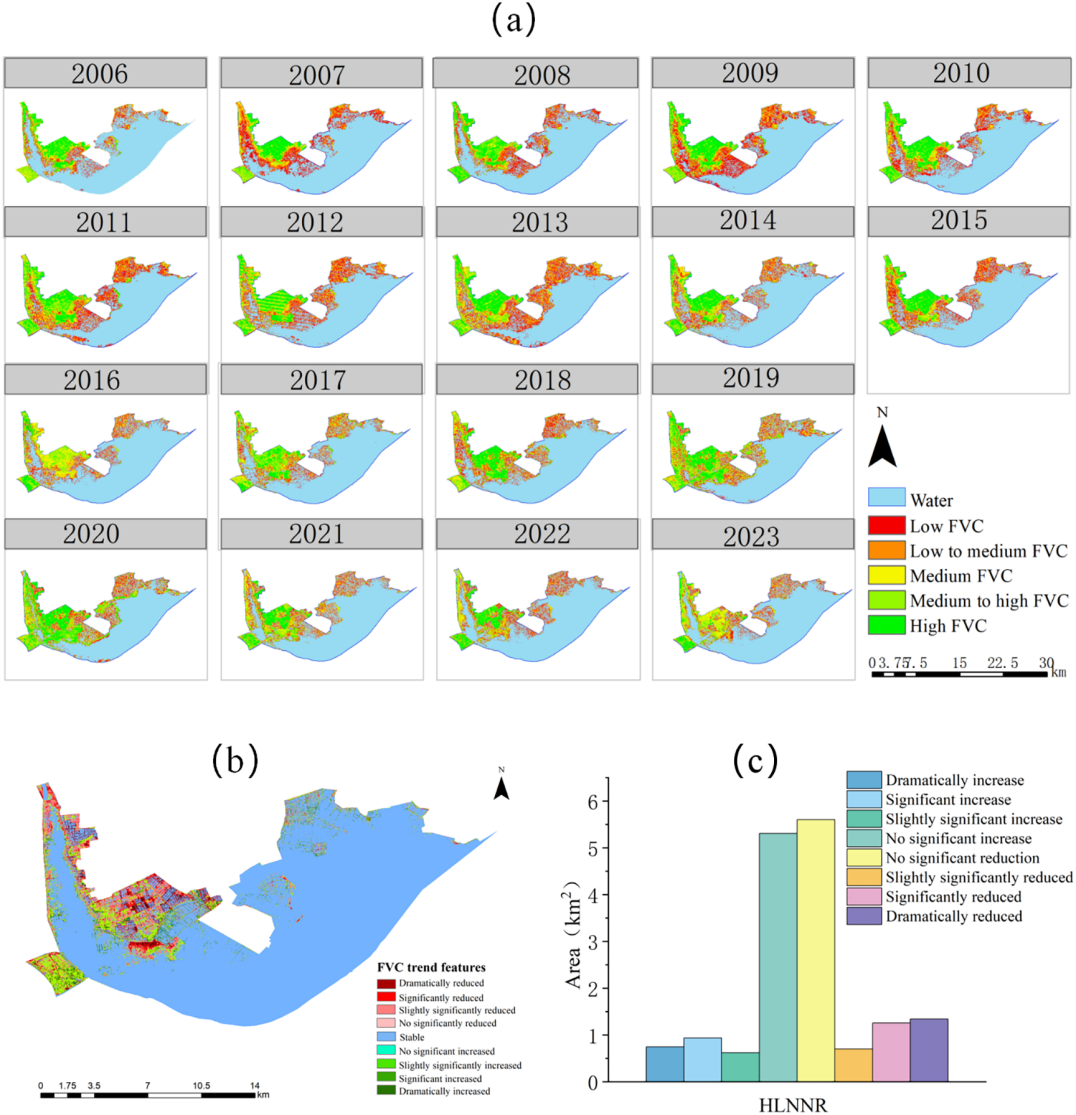
Map of spatial distribution of HLNNR **(A)** and trends in vegetation cover **(B)** and area statistics of trends in vegetation cover **(C)**, 20006-2023.

**Figure 8A** shows the changes in the spatial distribution of water area and aquatic vegetation cover in the HLNNR. Areas with high vegetation cover were mainly distributed in the northern part of the HLNNR. Areas with low vegetation cover were mainly distributed in the lakeshore zone, showing a decreasing pattern from the lakeshore zone to the open water zone. This indicates that the spatial distribution of aquatic vegetation in the HLNNR decreased from the land area to the water area. Aquatic vegetation cover had remarkable spatial heterogeneity. The ratio of aquatic vegetation to water area remained relatively stable throughout the study period (3:7). From 2006 to 2009, the overall aquatic vegetation increased with an increase in low vegetation cover from an overall area ratio of 5.57% to 11.39%. In addition, the overall water area decreased. In 2010–2016, 2018, 2021, and 2023, the vegetation cover was low and dominated by lower and low vegetation cover, that is, 11%–19% of the overall area ratio. In 2017, 2019, and 2020, the wetland areas were dominated by bare and waterless areas. Vegetation cover was high and dominated by higher vegetation cover, that is, 12% to 20% of the overall area ratio, with a sporadic distribution of aquatic vegetation.

The trend of vegetation cover changes in HLNNR obtained by applying a combination of Theil–Sen median trend analysis and the Mann–Kendall (MK) test is shown in **Figure 8B**. The distribution of aquatic vegetation along the waterside edges of the lakeshore zone is generally stable, mainly in areas with low water and vegetation cover. Vegetation in the northeastern portion of the HLNNR exhibited sporadic trends of minimal slightly significant to dramatic increases, with similar results in the portion of the HLNNR that protrudes from the southwest. Most of the vegetation intersecting the unaltered area showed slightly significant to dramatic increases. A large portion of the central to northern portion of the HLNNR showed a concentrated, slightly significant reduction or dramatic reduction.

**Figure 8C** and **Table 4** show that the overall vegetation area in the HLNNR area decreased slightly more than it increased during the study period. This indicates that the HLNNR area achieved a certain degree of success in the polder returning to the lake close to the watershed. However, there is still a lack of management of vegetation restoration near the center of the northern area.

**Table 4:** Percentage area values for each trend in vegetation cover.

Classification	Dramatic increased	Significant increased	Slightly significant increased	No significant increased	No significant reduced	Slightly significant reduced	Significant reduced	Dramatically reduced
Value	4.52%	5.67%	3.77%	32.13%	33.92%	4.26%	7.62%	8.12%

### 3.3 Spatial patterns and inter-annual variations in GPP and NPP cover

**Figure 9:**
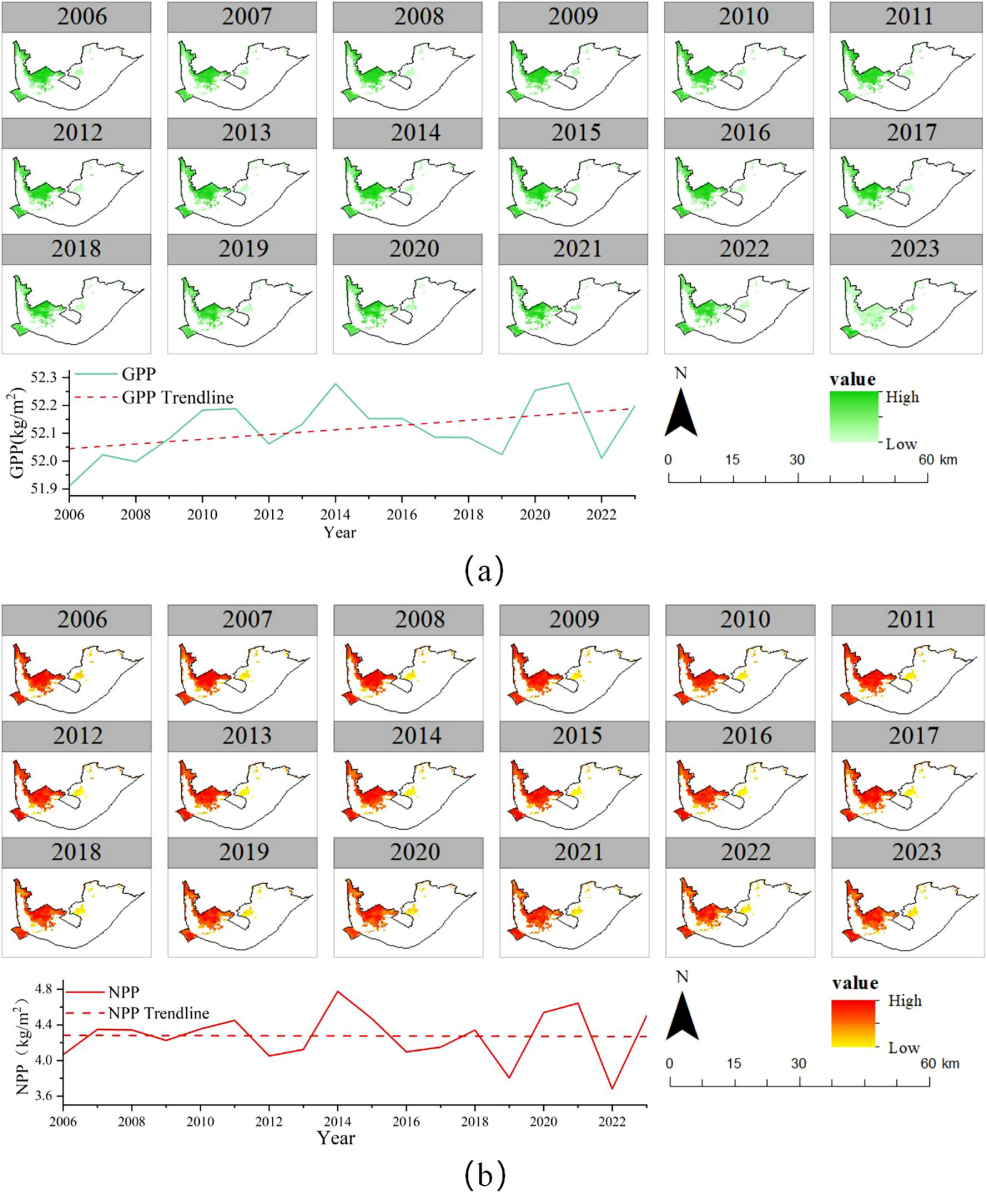
Spatial patterns and inter-annual variations in GPP **(A)** and NPP **(B)**.

GPP and NPP are important indicators of wetland carbon sinks. The annual average values of vegetation GPP and NPP in the HLNNR from 2006 to 2023 are shown in **Figures 9A, B**. Wetland vegetation GPP showed an overall increasing trend. The NPP showed an overall decreasing trend. The range of fluctuation was 51.91 kg/m^2^–52.28 kg/m^2^ and 3.68 kg/m^2^–4.78 kg/m^2^, respectively. The average GPP and NPP during the study period were 52.12 kg/m^2^ and 4.28 kg/m^2^, respectively. In 2021, the HLNNR annual mean vegetation GPP peaked at 52.28 kg/m^2^, 0.31% above the average. Meanwhile, the NPP peaked in 2014 at 4.78 kg/m^2^, 11.7% above the average. The minimum annual mean values of vegetation GPP and NPP of 51.91 kg/m^2^ and 3.68 kg/m^2^ occurred in 2008 and 2022, respectively. These values were 0.4% and 14% lower than the multi-year average, respectively. Overall, the fluctuations in the average values of vegetation GPP and NPP from 2006 to 2023 were small. The maximum increase or decrease did not exceed 0.52% and 20.7% of the average values, respectively.

According to **Figure 9**, the highest values for GPP and NPP in the HLNNR were mainly located in the northwestern part of the area where the vegetation is relatively lush. The low values for GPP and NPP were mainly located in the area where the water bodies met. They showed a decreasing pattern from the lakeshore zone to the open water zone, which has a high degree of spatial consistency with the distribution of aquatic vegetation. The spatial distributions of GPP and NPP showed a high level of consistency. The annual line graphs show that the overall GPP and NPP increased continuously, mainly near the northern side. From 2006 to 2009, the annual line graph shows that the overall GPP and NPP increased continuously. An increase in the area near the northern side was the main manifestation. As the proportion of aquatic vegetation and water areas in the HLNNR was relatively stable in 2010–2016, 2018, and in 2021–2023, the vegetation cover was relatively low. Difference in the spatial distribution of GPP and NPP was not significant. In 2017, 2019, and 2020, the HLNNR was dominated by the area of the water body. Therefore, the spatial distributions of GPP and NPP were scattered.

### 3.4 Correlation between areas of different vegetation cover and areas of water bodies

**Figure 10:**
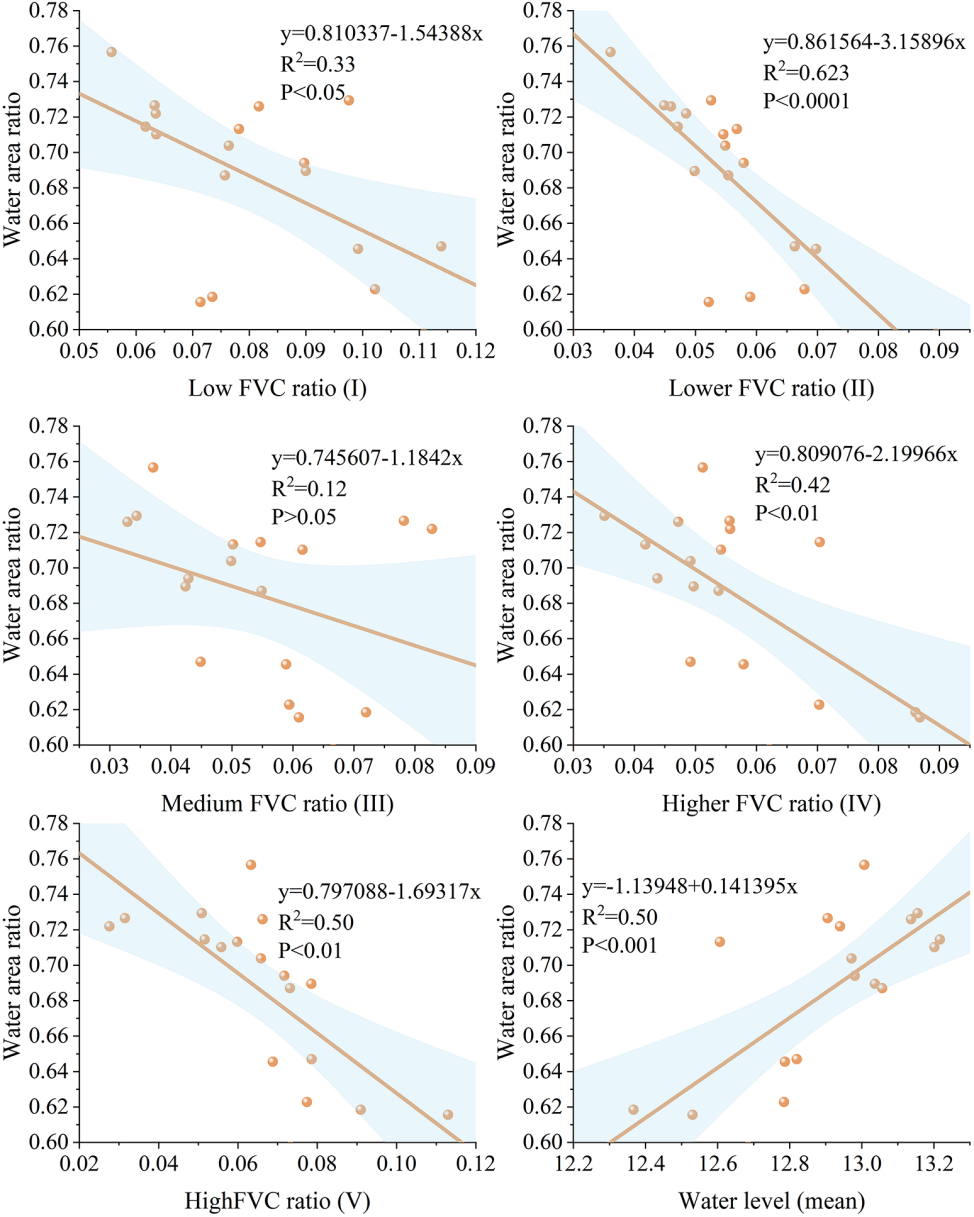
Correlation between the percentage of different aquatic vegetation cover in the study area and between the mean water level and the parameters of the percentage of water body area in the study area.

**Figure 10** shows the correlations among different aquatic vegetation cover ratios, mean water levels, and water body area parameters in the HLNNR. Except for the medium vegetation cover area ratio, the remaining vegetation cover area ratios showed a significant negative correlation (P < 0.05) with the water body area ratio. The correlation coefficients in descending order were as follow: lower FVC area ratio >high FVC area ratio > higher FVC area ratio > low FVC area ratio. The fluctuation of the water body had a greater impact on the vegetation of the lower vegetation cover area. This was due to the amplitude, frequency, duration, and regularity of lake WLF affecting the status of HLNNR aquatic vegetation and the allocation of water resources. The water level of Hongze Lake has the most remarkable effect on the area of water-supporting plants (Zhao et al., 2012). Therefore, the vegetation of lower FVC predominantly consists of water-supporting plants, and it is more susceptible to changes in lake level.

### 3.5 Correlation between different water level parameters, GPP, NPP, and vegetation cover

Fluctuations in the water body area affect different vegetation types to varying degrees. We analyzed the correlation between 11 different water level parameters of the wetland for GPP, NPP, and the area of different vegetation cover types. All the water level parameters, except max, WLF, L12.45, and L12.55, were significantly and positively correlated with NPP (P < 0.05). The correlation coefficients in descending order were as follows: L12.05 > 11.95 > L12.15 > L12.25 > min > mean > L12.35, and WLF was significantly negatively correlated with NPP (r = 0.59; P < 0.05). Except for max, min, and WLF, the different water level parameters showed significant negative correlation (P < 0.05) with the area of higher vegetation cover. The correlation coefficients, in descending order, were L12.35 = L12.45 > L12.55 > L12.25 > L12.15 > L11.95 > L12.05. The water level parameters mean and L12.45 showed the same significant correlation (r = 0.59; P < 0.05). The areas with high vegetation cover all showed the same significant negative correlation (r = −0.48; P < 0.05). The mean water level parameters and L12.35 were significantly negatively correlated (P < 0.05) with the mean area of cover, with correlation coefficients of r= −0.59 and r= −0.47, respectively.

## 4 Discussion

### 4.1 Effects of hydrologic conditions on wetland vegetation

WLF was the core factor affecting water allocation in the basin, and a key factor affecting the stability of wetland aquatic plant communities (Yang et al., 2023). The water level of Hongze Lake is subject to the combined effects of natural water inflow and artificial regulation. An anti-seasonal hydrological trend of low water levels in summer and high in winter has formed. Variations in the water level of Hongze Lake exhibit a strong correlation with the construction of water control gates and dams and anthropogenic water conservation projects (Zhao et al., 2012). Meanwhile, the eastern and southern parts of the HLNNR are mainly located in the center of the lake, with deeper water levels. The west and north belong to the shores of Hongze Lake, which has a shallow water level. In shallow water burial areas, the plant rhizosphere is in a state of long-term flooding or water saturation. The low soil oxygen content and poor aeration conditions of the water-logged soil habitat prevent plants not adapted to hygrophilous environments from surviving, resulting in only a limited number of plants to survive in the water-logged soil habitat (Zhu et al., 2013). Therefore, WLF can influence the vegetation patterns, characteristics, and ecological processes in the HLNNR, resulting in varying degrees of influence on different vegetation cover types (Liu et al., 2020). The wetland vegetation cover was generally higher when the wetland water body area decreased. This is in line with the findings of a previous study on the wetland of Dongting Lake based on Landsat observations of wetland changes over the past 40 years (Yang et al., 2020). When the area of the water bodies decreased rapidly, the wetland vegetation expanded significantly. Such natural rhythms are conducive to maintaining the long-term balance of aquatic ecosystems in the long-term (Hu et al., 2022).

**Figure 11:**
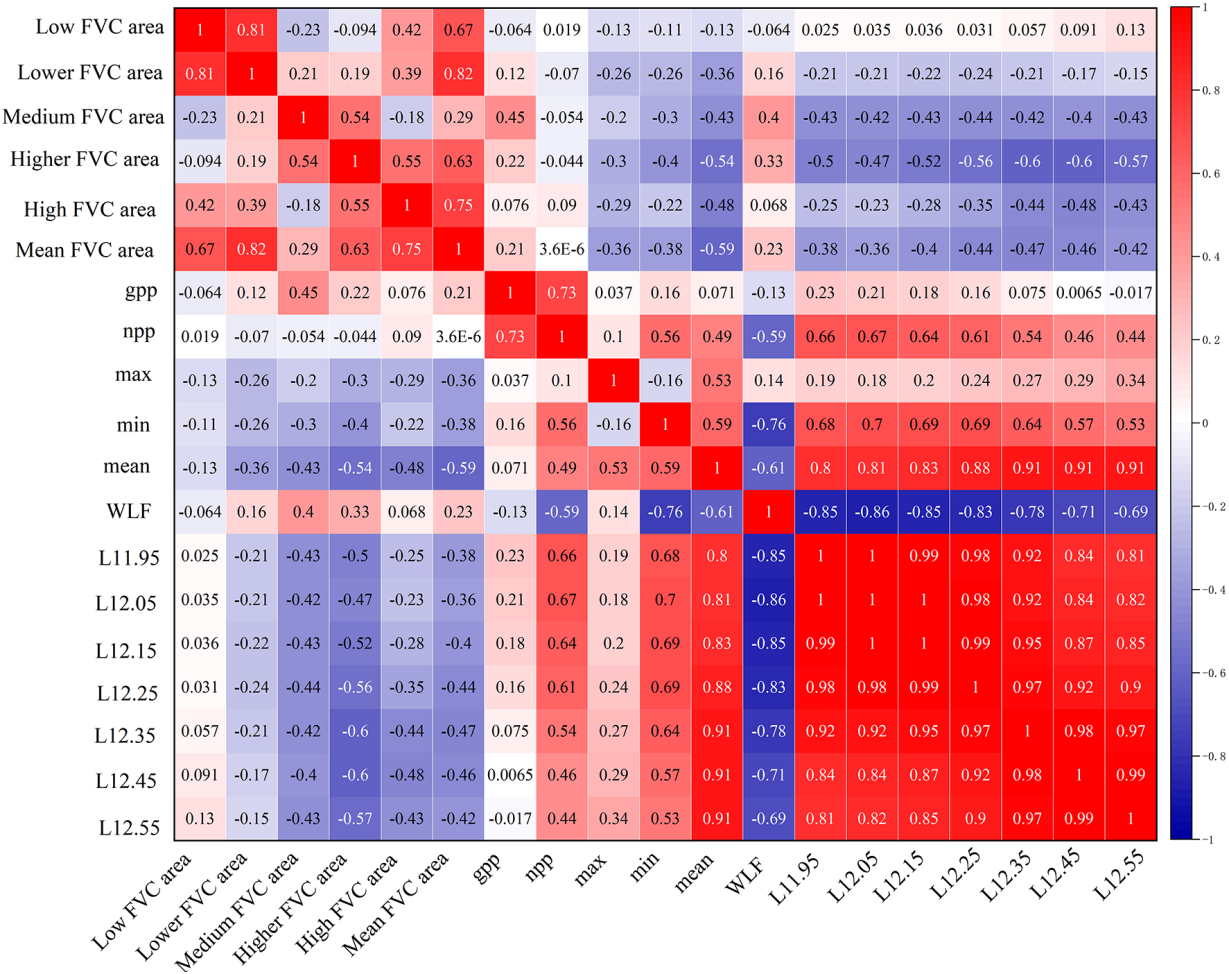
Correlation study of area ratio of different vegetation cover and GPP and NPP with indicators of annual water level change.

The extraction results of different FVC areas of the wetland for each year indicate a decrease in the total wetland vegetation area between 2006 and 2023. **Table 4** shows a decreasing trend in the overall area of incremental vegetation. By analyzing the water level thresholds affecting different vegetation covers in the wetlands of Hongze Lake, Pearson correlation analysis was conducted for different vegetation cover areas and different water level gradients, respectively (**Figure 11**). Different water level gradients were more sensitive to the effects of higher vegetation cover areas. The area of higher vegetation cover in Hongze Lake had a higher threshold for water level. After the water level reached 12.45 m, the negative correlation decreased. Conversely, when water levels are moderately high, the area of higher vegetation cover will increase, which contributes to the expansion of the distribution of higher vegetation cover.

### 4.2 How carbon sinks in lake wetlands are affected by water levels

The carbon sink characteristics of the East Dongting Lake watershed were investigated during the upwelling period and it was found that the net carbon sink flux (NPP) of the Dongting Lake watershed was mainly affected by CO_2_ flux, CO_2_ partial pressure, and dissolved oxygen (DO). Fluctuations in water level, water, temperature, and water alkalinity also have had a greater impact on the carbon sink capacity by altering the CO_2_ partial pressure content of the water (Zhu et al., 2023). This indirectly shows the existence of a large response of NPP to water level. During wetland restoration, suitable vegetation types can be selected according to the water level to enhance the wetland carbon sink function (Wang et al., 2024; Zhu et al., 2023; Jia et al., 2022). This indicates that the fluctuation in the water level affects the area of the water body and affects the type and area of vegetation. This, in turn, affects the vegetation carbon sink. Under certain conditions, owing to fluctuating water levels, temporary drying out of a lake or wetland area can change the site from a carbon sink to a carbon source. If a restoration program designs a bathymetric device to understand the deeper relationship between water levels and plants, this may lead to future maintenance issues, such as increasing water use to maintain adequate water levels and thus peak plant productivity and avoid drought stress (Valach et al., 2021). In this study, we quantified the annual inundation frequency of different water levels, obtained the area of different vegetation types, and analyzed their correlation. Except for the area of medium vegetation types, the other vegetation types were significantly negatively correlated with the water body area. We also analyzed the correlation of the annual inundation frequency of different water levels with the NPP and the GPP and found that the annual inundation frequency of the different water levels had a significant positive correlation with the NPP. However, the study area has higher topography in the northwest and lower topography in the southeast. When the water level reaches 11.95m, the flooding frequency of lakeshore zone is higher than that of other areas.This means that the lakeshore zone has a lower NPP. Areas with more flooding frequency and less vegetation have less carbon sink capacity (**Figure 9B**). The correlation coefficient of the lower water levels with the NPP was greater, indicating that the more inundated the wetland, the higher the carbon sink. The annual inundation frequency of different water levels had a significant positive correlation with NPP and the correlation coefficient between the lower water level and NPP was larger. Combined with the low northwest and high southeast water levels of the wetland, it was found that the larger the inundation range of the wetland, the higher the carbon sink of the wetland. This indicates that the carbon sink of the wetland is higher in the part of the wetland with higher inundation frequency. The aboveground vegetation carbon sink capacity is in line with that of the belowground carbon sink capacity. The carbon sink capacity of the soil increases with the frequency of inundation. This is because a higher water table reduces the soil oxygen availability, thus limiting soil respiration. This leads to lower atmospheric fluxes of carbon dioxide (Regier et al., 2023; Xiong et al., 2023; Evans et al., 2021; Schaufler et al., 2010).

A certain amount of peat carbon is present in the central to northwestern portion of the HLNNR region (Lu and Xu 2014). Peat carbon accumulation in wetlands is controlled by large-scale factors, such as climate and hydrological environment, and small-scale factors, such as microtopography and plant communities (Mathijssen et al., 2019). In revegetated wetlands, the pattern of aboveground biomass succession results in lower biodiversity, especially in exposed low vegetation wetlands that are more susceptible to environmental impacts (Mitsch et al., 2005). Soil carbon in wetlands is largely protected by anaerobic conditions when disturbances involving changes in water levels increase respiration (Resp) and decrease NPP. This, in turn, may lead to disproportionately large carbon losses, as observed in other disturbed wetlands (Macreadie et al., 2013; Moomaw et al., 2018). When feasible, efforts are made to regulate the water levels in wetlands to mitigate the effects of extreme events on NPP. Consequently, this aids in maintaining high carbon sequestration and minimizing respiration. This is dependent on the design of the wetland water level, which may include keeping the water level fully submerged in the soil while protecting plants from salt invasion and accumulation, pests, and pathogen infections in freshwater systems (Valach et al., 2021). In contrast, the wetlands in this study had substantial water level variations and complex change scenarios, underscoring the importance of effectively managing the water levels. In conjunction with the data presented in **Figures 8B**, **9A** and **11B**, as well as the topography of the HLNNR area (a trend of decreasing vegetation in the northern part of the FVC and an increasing trend in the lakeshore zone, as well as the fact that vegetation in the lakeshore zone tends to be of low to lower FVC and that the response of the lower FVC to the water body area is the most pronounced), the net carbon sequestration capacity of the vegetation is greater in the HLNNR area, but wetland areas with lower FVC and lower carbon sequestration are vulnerable to water level disturbance events. We therefore emphasize the importance and necessity of considering water levels for these specific sites in wetland restoration design and post-restoration management.

### 4.3 Uncertainties and limitations

This study focuses on the fluctuations in water levels and water bodies and their impact on aquatic vegetation and provides a valuable reference for wetland restoration design and post-restoration water level management in the HLNNR area. Water quality, current flow rate, clarity, and water temperature have potential effects on aquatic vegetation. These factors may also have played a role in influencing the results of this study. Further locational, long-term, and controlled tests are required to validate these results. In addition, another objective of this study was to investigate the effect of hydrologic changes on GPP and NPP in the HLNNR area. Although vegetation carbon storage in lake and wetland systems is affected by multiple factors, precipitation and temperature-induced changes in water levels play a crucial role in shaping the overall dynamics of wetland ecosystems. Temperature is the is the predominant factor impacting the fluctuation of soil and sediment carbon content, which determines the carbon source and sink status of wetland ecosystems. In reality, it is difficult to fully distinguish between the effects of climate change and human activities on vegetation GPP and NPP, which inevitably have an impact on the optimization and validation of the results. Thus, further studies are required on the interactions between hydrological-related processes and vegetation GPP and NPP under the influence of climatic regulation.

## 5 Conclusions

In this study, the relative changes and trends in FVC, GPP, NPP, and water surface area in Hongze Lake from 2006 to 2023 were analyzed using the Google Earth Engine (GEE) platform. The daily water level data were collected from the hydrological Station to study the impact of water level fluctuations (WLF) on the above parameters. The main findings are as follows:

1. Significant variability in vegetation distribution. The spatial distribution of vegetation in the Hongze Lake wetland varied considerably. It showed a transition pattern from high to low vegetation coverage from the land areas to water areas. This was primarily driven by the significant water level fluctuations, and the minimal changes in waterbody extent due to topographic characteristics. Consequently, the vegetation cover in this area was almost entirely influenced by hydrological factors.
2. Higher carbon sequestration with increased inundation frequency. Wetland areas of Hongze Lake with higher inundation frequencies acted as greater carbon sinks. The FVC in the lakeshore zone was on the rise, and there was a significant positive correlation (P < 0.05) between NPP and annual water level frequency.
3. Positive outcomes from ecological restoration. The implementation of the “returning farmland to lake” project in Hongze Lake has yielded positive results, contributing to increased carbon sequestration in the wetland vegetation. While the overall FVC area showed a decreasing trend, it was increasing in the lakeshore zone. The higher inundation frequency in this zone has further enhanced carbon sequestration in the wetland vegetation.

These findings are crucial for understanding the potential driving mechanisms of carbon cycling and aquatic ecosystem functions in seasonal lake wetlands. They highlights the importance of water level management in restoring vegetation carbon sinks in lake wetlands and provide new insights into carbon cycling and ecosystem functionality in these environments.

## Data Availability

Datasets are available on request. The raw data supporting the conclusions of this article will be made available by the authors, without undue reservation.
